# Analysis of cumulative live birth rate and perinatal outcomes in young patients with low anti-müllerian hormone levels using two ovulation promotion protocols: A cohort study

**DOI:** 10.3389/fendo.2022.938500

**Published:** 2022-08-05

**Authors:** Zhen Li, Ruolin Jia, Kexin Wang, Junwei Zhang, Bingnan Ren, Yichun Guan

**Affiliations:** The Reproduction Center, The Third Affiliated Hospital of Zhengzhou University, Zhengzhou, China

**Keywords:** anti-Mullerian hormone, *in vitro* fertilization/intracytoplasmic single sperm injection, GnRH antagonist protocol, progestin-primed ovarian stimulation protocol, cumulative live birth rate

## Abstract

**Objective:**

To compare cumulative live birth rates and perinatal outcomes of young IVF/ICSI patients with low anti-Mullerian hormone (AMH) levels on a gonadotropin-releasing hormone antagonist (GnRH-ant) regimen with those on a high progesterone state of ovulation (PPOS) regimen.

**Methods:**

We retrospectively analyzed 798 patients who underwent *in vitro* fertilization (IVF) or intracytoplasmic sperm microinjection (ICSI) between January 2015 and December 2020 at the Third Affiliated Hospital of Zhengzhou University. A total of 798 cycles of complete clinical data from patients who underwent *in vitro* fertilization (IVF) or intracytoplasmic sperm injection (ICSI) at the Reproductive Medicine Center of Zhengzhou University Hospital between January 2015 and December 2020 and were eligible for AMH < 1.2 ng/ml at age < 35 years, Group A1: very low AMH levels (AMH < 0.5 ng/mL) and GnRH antagonist regimen; Group A2, very low AMH level (AMH < 0.5 ng/mL) and PPOS regimen; Group B1, low AMH level (0.5 ng/mL ≤ AMH < 1.2 ng/mL) and GnRH antagonist regimen; and Group B2, low AMH level (0.5 ng/mL ≤ AMH < 1.2 ng/mL), and the PPOS regimen.

**Results:**

At very low levels of AMH (< 0.5 ng/mL), the CLBR of the GnRH antagonist regimen was not significantly different from that of the PPOS regimen (P > 0.05), at 0.5 ng/mL ≤ AMH < 1.2 ng/mL. Statistics showed that the CLBR of the GnRH antagonist regimen was significantly higher than that of the PPOS regimen (49.7% vs. 35.7%, P=0.002). Logistic regression analysis showed that in Group A: the younger the female partner, the higher the CLBR (OR = 0.972, 95% CI = 0.923–1.042, P = 0.022), and the more the AFC, the higher the CLBR (OR = 1.166, 95% CI = 1.091–1.336, P < 0.001). Group B: the higher the number of good-quality embryos, the higher the CLBR (OR = 2.227, 95% CI = 1.869–2.654, P < 0.001). Compared with PPOS regimens, the antagonist regimen was able to increase the CLBR. The analysis of Group A showed that the antagonist regimen had a shorter TTP than the PPOS regimen (P < 0.001); however, the PPOS regimen had a lower cost of ovulation (4311.91 vs. 4903.81, P = 0.023). The antagonist regimen in Group B had a shorter TTP than the PPOS regimen, and there was no significant difference in the cost of ovulation. In the analysis of perinatal outcomes, there were no statistically significant differences in preterm birth, low birth weight, very low birth weight, and pregnancy complications among the four groups.

**Conclusion:**

Young patients with very low AMH levels (< 0. 5 ng/mL), the GnRH antagonist regimen was comparable to the PPOS regimen in CLBR outcomes; the antagonist regimen shortens the time to clinical pregnancy, and the PPOS regimen is more cost-effective. In young patients with low AMH levels of 0.5 ng/mL and <1.2 ng/mL, the GnRH antagonist regimen can more appropriate to improve CLBR, and the perinatal outcomes were similar for both regimens.

## 1 Introduction

In assisted reproductive technology (ART)-assisted conception, the dose-response relationship between serum AMH and oocyte acquisition and its accuracy in predicting poor and excessive responses to ovarian stimulation has been well established (1). Poor ovarian response (POR) during controlled ovarian stimulation is one of the most challenging problems for reproductive clinicians (2). POR is often characterized by low AMH levels and low numbers of oocytes obtained using ovarian stimulation protocols. Owing to the low number of eggs obtained, patients with POR have higher cycle cancellation rates, lower pregnancy rates, and a lower cumulative live birth rate per starting cycle than normal responders (3). In many fertility centers, antagonist regimens and progestin-primed ovarian stimulation (PPOS) regimens are becoming ovulation regimens that are commonly used for patients with low ovarian response (4, 5). Research on POR has focused on the use of progesterone in the ovarian system. The literature on POR has focused on women aged > 35 years because of the age-related decline in ovarian reserve function. However, we have found that the incidence of POR in women aged under 35 years is similar to that in women older than 35 years (6). In this study, we performed a retrospective analysis of patients who underwent IVF/ICSI in the last 5 years at our center and were of age < 35 years with AMH < 1.2 ng/ml to explore the possible impact of different ovulation regimens on the final pregnancy outcome of IVF/ICSI in young patients with low AMH levels, to select the best ovulation regimen for young patients with different levels of low AMH in clinical practice. The results of this review are used to examine the possible impact of ovulation regimens on the final pregnancy outcome of young patients with different levels of low AMH and to improve the pregnancy outcome of patients with POR during ART.

## 2 Materials and methods

### 2.1 Study design and population

Patients who underwent *in vitro* fertilization (IVF) or intracytoplasmic sperm injection (ICSI) at the Department of Reproductive Medicine of the Third Affiliated Hospital of Zhengzhou University between January 2015 and December 2020 were selected for the retrospective analysis. The inclusion criteria were: 1) age < 35 years, 2) AMH < 1.2 ng/ml (= 8.568pmol/L), and 3) use of a gonadotropin-releasing hormone antagonist (GnRH-ant) regimen or a high progesterone state pro-ovulation (PPOS) regimen. The exclusion criteria were: 1) endometrial polyps, uterine adhesions, or abnormal uterine anatomy; 2) endocrine disorders namely, abnormal thyroid function, hyperprolactinemia; 3) systemic diseases such as reproductive tuberculosis; 4) women undergoing PGT and women with known chromosomal abnormalities; and 5) frozen oocytes or oocytes obtained through donation.

In total, 798 cycles of complete clinical data were available. Grouping was conducted according to the participants’ AMH levels and different ovulation regimens: Group A1: very low AMH levels (AMH < 0.5 ng/mL) and antagonist regimens for 54 cycles; Group A2, very low AMH levels (AMH < 0.5 ng/mL) and PPOS regimens, 214 cycles; Group B1: low AMH levels (0. 5 ng/mL ≤ AMH < 1.2 ng/mL) and antagonist regimens for 191 cycles; Group B2: low AMH levels (0. 5 ng/mL ≤ AMH < 1.2 ng/mL) and PPOS regimen groups,339 cycles.

### 2.2 Testing

(1) Blood AMH test: 4 mL of blood were drawn on empty stomach and sent to the test center for chemiluminescence testing.

(2) Sex hormones (follicle-stimulating hormone (FSH), luteinizing hormone (LH), estradiol (E2)): Venous blood (4 mL) was collected at 2–4 d of the menstrual cycle on empty stomach and assayed by electrochemiluminescence. The assay was performed by electrochemiluminescence using an E602 assay device, and the kit was purchased from the manufacturer.

(3) AFC test: The same vaginal ultrasound machine was used to measure sinus follicles—2–10 mm in diameter at 2–4 d of the menstrual cycle—and the total number of follicles in both ovaries was calculated.

### 2.3 Controlled ovarian hyperstimulation protocols

#### 2.3.1 GnRH antagonist protocol

In the GnRH antagonist group, initiation was started on days 2 and 3 of the menstrual cycle, combining patient age, body mass index (BMI), AMH, basal FSH levels, and basal AFC, with a starting dose of Gn of 150–300 IU/d, added when the mean follicle diameter was 11–12 mm and the estradiol was > 500 ng/L (1835 pmol/L) GnRH-A (Stryker, Merck Serono, USA) or Ganirelix acetate injection, (N.V. Organon, The Netherlands), triggered when three follicles were > 17 mm in diameter or two follicles were >18 mm in diameter. Trigger drug selection: if women who were OHSS-prone, they can choose the plan A:GnRH-a [Dabigat, Pfizer (Switzerland) Pharma Ltd] 0.2 mg trigger + all embryos frozen, 33–36 h later puncture for egg retrieval under vaginal ultrasound guidance; for women who were not OHSS-prone, they can choose the plan B:Azer 250 μg trigger, and then 36 h later egg retrieval + fresh cycle transplantation. Conventional IVF/ICSI insemination was performed.

#### 2.3.2 PPOS

For the PPOS regimen, a Gn initiation dose from 75–300 IU/day was administered on days 2 and 3 of the menstrual cycle, depending on the patient’s age, body mass, BMI, AMH, and basal AFC, along with 6 mg medroxyprogesterone acetate (MPA) (Zhongxin Pharmaceuticals, Beijing, China). Follicle growth was monitored using vaginal ultrasound combined with serum hormone analysis after 4 d. If necessary, the dose of Gn was adjusted according to the follicle development. When three follicles were > 17 mm in diameter or two follicles were >18 mm in diameter, the final stage of ovulation triggering was performed with Daphne (Epsom, France) 0.1 mg and 2000 IU human chorionic gonadotropin (hCG) (Zhuhai Lizhu Group Lizhu Pharmaceuticals), followed by egg retrieval at 36 h.

### 2.4 Embryo transfer and endometrial preparation protocols

For patients on the antagonist regimen, fresh cycle transfer was possible if the endometrium was in good condition (thickness ≥ 7 mm; acceptable morphology) and there were no contraindications to transfer. Whole embryo freezing was performed in patients treated with PPOS.

Depending on the regularity of the menstrual cycle, a natural cycle or hormone replacement cycle was routinely selected to prepare the endometrium. Stimulation cycles were used if the menstrual cycle was regular, but follicular development was monitored using ovulation-promoting drugs.

### 2.5 Outcome measures and definition

The primary observation was the cumulative live birth rate (CLBR). The secondary observation was TTP: the time from the start of the prolotherapy program to clinical pregnancy. A clinical pregnancy rate [(number of clinical pregnancy cycles/number of transplant cycles) × 100%] determined by a positive blood test for β-hCG 14 d after transplantation was considered a pregnancy; a clinical pregnancy was diagnosed on an ultrasound 30 d after transplantation if a gestational sac was observed. Perinatal indicators: Gestational age (GA) is defined as the age from the first day of the last normal menstrual period to the time of delivery, usually expressed in weeks, for preterm babies (newborns with a GA < 37 weeks). The birth weight of newborns born alive was assessed as follows: low birth weight (LBW < 2500 g) and very low birth weight (VLBW < 1500 g) (7). Common pregnancy complications of IVF/ICSI comprised gestational hypertension, gestational diabetes, placenta previa, and premature membrane rupture (8, 9).

### 2.6 Assessment of CLBR

(1) CLBR: the number of patients who obtained a live birth in a fresh cycle after ovarian stimulation and the subsequent thawing transfer cycle/number of patients who started an ovarian stimulation cycle; (2) Optimistic CLBR: assumes that women who do not return for a subsequent IVF cycle have the same chance of becoming pregnant as those who return for treatment; (3) Conservative CLBR: assumes that patients who do not return for subsequent IVF/ICSI treatment have no chance of a pregnancy resulting in a live birth (10, 11).

### 2.7 Statistical analysis

Statistical analysis was performed using SPSS 24.0, with normally distributed results expressed as mean ± standard deviation (mean ± SD); non-normally distributed measures were expressed as median (25th percentile, 75th percentile) [M(Q1, Q3)]; quantitative data between the two groups were tested using the rank sum test or independent samples *t* test; count data were expressed as frequency (%) using the χ2 test, and the factors influencing the CLBR were analyzed using logistic regression analysis. The test level was set at P < 0.05.

## 3 Results

A total of 798 egg retrieval cycles were included in this study, with 245 cycles in the antagonist group and 553 cycles in the PPOS group.

### 3.1 Baseline characteristics

In the antagonist regimen for young patients, the AMH level,AFC, Gn usage time, E2 level on HCG day, the total number of eggs obtained, 2PN number, number of available embryos, number of high-quality embryos and the cumulative live birth rate in group B1 were higher than those in group A1 (P<0.05). Details are provided in **Table 1**.

**Table 1:** Comparison of basic clinical information and laboratory results of patients with different AMH levels using antagonist regimens.

Projects	Antagonist regimen (A1)	Antagonist regimen (B1)	Z/t/*x²* Value	P-value
Number of cycles	54	191		
Female age (years)	30 (28, 32)	31.5 (29, 33)	0.374	0.709
Body mass index (kg/m2)	23.92 ± 0.27	23.68 ± 0.26	-0.732	0.465
Years of infertility (years)	3 (2, 5)	3(2,5)	0.007	0.995
Anti-Mullerian hormone (ng/ml)	0.32 (0.24, 0.42)	0.85(0.71,1.00)	-20.060	<0.001
Basal FSH (IU/L)	10(7,16)	8.32 (6.04, 9.58)	4.438	<0.001
Basal E2 (pmol/L)	160.2 (114.4, 193.8)	159.05 (119.96, 192.84)	-0.390	0.697
Sinus follicle count AFC	6(4,7)	8(6,11)	-5.756	<0.001
Gn initiation volume (U)	300.0(225.0, 300.0)	300 (225, 300)	-1.759	0.08
Gn usage time (d)	9 (7.25, 10)	10 (8, 11)	-2.208	0.028
hCG Day				
The endometrial thickness on hCG day (mm)	10.19 ± 0.33	10.36 ± 0.17	-0.770	0.442
hCG day LH (IU/L)	3.725 (2.03, 6.27)	1.8 (1.1125, 2.975)	5.071	<0.001
hCG day estradiol (pmol/L)	2171.5 (898.25, 3991.0)	3362 (1582, 6232.5)	-4.043	<0.001
Total number of eggs obtained (pieces)	3(2,4.75)	6 (4, 9)	-6.019	<0.001
2PN number (pieces)	2(1,4)	3 (2, 5)	-4.166	<0.001
Number of available embryos (pieces)	2(1,3)	3 (2, 4)	-4.231	<0.001
Number of high-quality embryos (pieces)	1 (0, 1)	1 (0, 2)	-3.140	<0.001
Cumulative live birth rate	22.2% (12/54)	49.7% (95/191)	12.957	<0.001
Optimistic cumulative live birth rate	22.0% (13/59)	49.3% (110/223)	14.133	<0.001
Conservative cumulative live birth rate	20.3% (12/59)	42.6% (95/223)	9.820	0.002

In the PPOS regimen for young patients, The AMH level, AFC, Gn initiation dose, E2 level on HCG day, the total number of eggs obtained, 2PN number, number of available embryos, number of high-quality embryos and the cumulative live birth rate in group B2 were higher than those in group A2 (P<0.05). Details are provided in **Table 2**.

**Table 2:** Comparison of basic clinical information and laboratory results of patients with different AMH levels using PPOS protocol.

Projects	PPOS Program (A2)	PPOS Program (B2)	Z/t/*x²* Value	P-value
Number of cycles	214	339		
Female age (years)	31 (29, 33)	31 (29, 33)	-0.312	0.755
Body mass index (kg/m2)	23.00 ± 0.46	23.53 ± 0.19	1.706	0.089
Years of infertility (years)	3 (2, 6)	3(2,5)	0.255	0.799
Anti-Mullerian hormone (ng/ml)	0.32 (0.22, 0.43)	0.82(0.68,0.99)	-15.826	<0.001
Basal FSH (IU/L)	9(7,14)	8.37 (6.60, 10.71)	-0.178	0.859
Basal E2 (pmol/L)	138.8 (86.4, 200.4)	146.4 (104.35,196.4)	0.470	0.639
Sinus follicle count AFC	4(3,6)	7(5,10)	-9.137	<0.001
Gn initiation volume (U)	300.0(225.0, 300.0)	300 (300, 300)	-4.447	<0.001
Gn usage time (d)	9 (8.0, 12.0)	9 (8, 11)	1.623	0.105
hCG Day				
The endometrial thickness on hCG day (mm)	7.63 ± 0.15	7.93 ± 0.11	-0.631	0.528
hCG day LH (IU/L)	3.61 (2.01, 5.10)	3.035 (1.81, 4.608)	3.695	<0.001
hCG day estradiol (pmol/L)	2859.0 (1499.0, 4581.0)	5065(3319.5,7478.5)	-7.898	<0.001
Total number of eggs obtained (pieces)	3(1,5)	5 (3, 7)	-7.944	<0.001
2PN number (pieces)	2(1,3)	3 (2, 5)	-5.804	<0.001
Number of available embryos (pieces)	2(1,2)	2 (1, 4)	-4.807	<0.001
Number of high-quality embryos (pieces)	1 (0, 2)	1 (0, 3)	-4.507	<0.001
Cumulative live birth rate	26.6% (57/214)	35.7% (121/339)	4.931	0.026
Optimistic cumulative live birth rate	26.5% (69/260)	35.6% (154/432)	6.167	0.013
Conservative cumulative live birth rate	21.9% (57/260)	28.0% (121/432)	3.147	0.076

Groups A1 and A2 showed no statistical difference in BMI, years of infertility, basal FSH, Gn initiation, total number of eggs gained, number of 2PN, number of available embryos, and number of good-quality embryos (p > 0.05). Patients on the PPOS regimen had a higher duration of Gn use than those on the antagonist regimen and a lower number of sinus follicles than those on the antagonist regimen. Details are provided in **Table 3**.

**Table 3:** Group A patients Comparison of basic clinical information and laboratory results [*xˉ ± s*, M(Q1, Q3)].

Projects	Antagonist regimen (A1)	PPOS Program (A2)	*Z/t/ x²* values	*P* value
Number of cycles	54	214		
Age of woman (years)	30 (28, 32)	31 (29, 33)	2.419	0.016
Body mass index (kg/m^2^)	23.92 ± 0.27	23.00 ± 0.46	1.592	0.113
Years of infertility (years)	3 (2, 5)	3 (2, 6)	-0.219	0.827
Anti-Mullerian hormone (ng/ml)	0.32 (0.24, 0.42)	0.32 (0.22, 0.43)	-0.187	0.851
Basal FSH (IU/L)	10(7,16)	9(7,14)	-1.094	0.274
Basal E2 (pmol/L)	160.2 (114.4, 193.8)	138.8 (86.4, 200.4)	-1.397	0.163
Sinus follicle count AFC	6(4,7)	4(3,6)	-2.659	0.036
Gn initiation volume (U)	300.0 (225.0, 300.0)	300.0 (225.0, 300.0)	-1.388	0.165
Gn Duration of use (d)	9 (7.25, 10)	9 (8.0, 12.0)	-2.607	0.040
hCG Day				
hCG day endometrial thickness (mm)	10.19 ± 0.33	7.63 ± 0.15	-7.493	<0.001
hCG day LH (IU/L)	3.725 (2.03, 6.27)	3.61 (2.01, 5.10)	-0.468	0.64
hCG day estradiol (pmol/L)	2171.5 (898.25, 3991.0)	2859.0 (1499.0, 4581.0)	-2.012	0.047
Total number of eggs harvested (pieces)	3(2,4.75)	3(1,5)	-0.684	0.494
2PN number (pieces)	2(1,4)	2(1,3)	-0.350	0.726
Number of embryos available (pieces)	2(1,3)	2(1,2)	-0.253	0.800
Number of good-quality embryos (pieces)	1 (0, 1)	1 (0, 2)	-0.422	0.673
Cumulative live birth rate	22.2% (12/54)	26.6% (57/214)	0.439	0.507
Optimistic cumulative live birth rate	22.0% (13/59)	26.5% (69/260)	0.511	0.475
Conservative cumulative live birth rate	20.3% (12/59)	21.9% (57/260)	0.071	0.790

A comparison of the basic clinical data and laboratory results of patients in groups B1 and B2 showed no statistically significant differences in age, BMI, or years of infertility, and the PPOS regimen had a higher Gn initiation than the antagonist regimen. Details are provided in **Table 4**.

**Table 4:** Comparison of basic clinical information and laboratory results of patients in Group B [xˉ ± s, M(Q1, Q3)].

Projects	Antagonist regimen (B1)	PPOS Program (B2)	*Z/t/x²* values	*P* value
Number of cycles	191	339		
Age of woman (years)	31.5 (29, 33)	31 (29, 33)	0.266	0.790
Body mass index (kg/m^2^)	23.68 ± 0.26	23.53 ± 0.19	0.481	0.631
Years of infertility (years)	3(2,5)	3(2,5)	-0.491	0.623
Anti-Mullerian hormone (ng/ml)	0.85(0.71,1.00)	0.82(0.68,0.99)	-1.167	0.243
Basal FSH (IU/L)	8.32 (6.04, 9.58)	8.37 (6.60, 10.71)	-1.774	0.201
Basal E2 (pmol/L)	159.05 (119.96, 192.84)	146.4 (104.35,196.4)	-1.639	0.101
Sinus follicle count AFC	8(6,11)	7(5,10)	-4.119	<0.001
Gn initiation volume (U)	300 (225, 300)	300 (300, 300)	-6.558	<0.001
Gn Duration of use (d)	10 (8, 11)	9 (8, 11)	-0.914	0.361
hCG Day				
hCG day endometrial thickness (mm)	10.36 ± 0.17	7.93 ± 0.11	12.329	<0.001
hCG day LH (IU/L)	1.8 (1.1125, 2.975)	3.035 (1.81, 4.608)	-6.516	<0.001
hCG day estradiol (pmol/L)	3362 (1582, 6232.5)	5065 (3319.5,7478.5)	-5.373	<0.001
Total number of eggs harvested (pieces)	6 (4, 9)	5 (3, 7)	-3.284	0.001
2PN number (pieces)	3 (2, 5)	3 (2, 5)	-1.772	0.085
Number of embryos available (pieces)	3 (2, 4)	2 (1, 4)	-2.094	0.036
Number of good-quality embryos (pieces)	1 (0, 2)	1 (0, 3)	-0.413	0.680
Cumulative live birth rate	49.7% (95/191)	35.7% (121/339)	9.981	0.002
Optimistic cumulative live birth rate	49.3% (110/223)	35.6% (154/432)	11.439	<0.001
Conservative cumulative live birth rate	42.6% (95/223)	28.0% (121/432)	14.168	<0.001

### 3.2 Factors associated with CLBR

There was no significant difference in CLBR between the two regimens for patients with very low AMH levels, and the choice of antagonist regimen for patients with low AMH levels can improve the cumulative live birth rate (**Tables 3**, **4**). To explore the possible factors affecting CLBR, we performed multiple regression analysis on clinical indicators such as female’s partner’s age, AFC, AMH, female BMI, number of good-quality embryos, and the two prolotherapy regimens. As shown in **Table 5**, Group A: the younger the female’s partner, the higher the CLBR (OR = 0.972, 95% CI = 0.923–1.042, P = 0.022), and the more the AFC (OR = 1.166, 95% CI = 1.091–1.336, P < 0.001), while the higher the number of good-quality embryos, the higher the CLBR (OR = 2.654, 95% CI = 1.911–3.687, P < 0.001). Group B: the higher the number of good-quality embryos, the higher the CLBR (OR = 2.227, 95% CI = ~1.869–2.654, P < 0.001), the antagonist regimen was able to increase CLBR compared with PPOS regimens (OR = 0.499, 95% CI = 0.324–0.767, P = 0.002 for the PPOS regimen with the antagonist regimen as the control group).

**Table 5:** Multi-factor logistic analysis of cumulative live birth rate.

Variables	*B*	Wald	*OR* value	95% CI	*P* value
**Group A**					
Age of woman	0.159	0.237	0.972	0.923~1.042	0.022
AFC	0.153	6.368	1.166	1.091~1.336	< 0.001
AMH	0.047	0.002	1.048	0.102~10.806	0.969
BMI	0.077	2.701	1.080	0.985~1.183	0.100
Number of good-quality embryos	0.976	33.894	2.654	1.911~3.687	<0.001
Ovulation Program					
Antagonists	-0.190	0.197	0.827	0.358~1.913	0.657
PPOS (control group)	–	–	–	–	–
**Group B**					
Age of woman	0.003	0.008	1.003	0.933~1.079	0.928
AFC	0.022	0.491	1.022	0.961~1.088	0.484
AMH	0.320	0.322	1.378	0.455~4.169	0.570
BMI	-0.018	0.337	0.982	0.924~1.044	0.562
Number of good-quality embryos	0.801	80.142	2.227	1.869~2.654	< 0.001
Ovulation Program#					
Antagonist (control group)	–	–	–	–	–
PPOS	-0.696	10.044	0.499	0.324~0.767	0.002

#Referenced by the antagonist protocol.

### 3.3 Comparison of patients’ TTP and prolotherapy costs

As shown in **Table 6**, the antagonist regimen in Group A had a significantly higher cost of promotion during ovulation than the PPOS regimen, and the antagonist regimen had a significantly lower TTP than the PPOS regimen (p < 0.001). The rate of embryo-free cycle transfer was not statistically different between the two regimens at very low AMH levels (*P > 0.05*), and the rate of embryo-free cycle transfer was higher for the PPOS regimen than for the antagonist regimen in Group B (19.2% *vs.* 2.1%, *P < 0.001*); the clinical pregnancy rate was higher for patients using the antagonist regimen than the PPOS regimen.

**Table 6:** Comparison of patient cost-effectiveness and TTP.

Projects	Antagonist regimen (1)	PPOS program (2)	*t/Z/x^2* values	*P* value
Group A				
Cycle TTP(d)*				
Fresh transplant cycle	41.11 ± 7.88	–		
Freeze-thaw transplant cycle	122.41 ± 36.42	177.94 ± 39.67	4.039	<0.001
Total TTP	116.56 ± 11.52	177.94 ± 39.67	-1.601	0.008
Embryo-free transfer cycle rate (%)	24.1 (13/54)	29.0 (62/214)	0.513	0.474
Cost of ovulation promotion treatment ($)	4903.81 (3444.84, 5905.44)	4311.91 (1101.75, 5905.44)	-2.275	0.023
Group B				
Cycle TTP(d)*				
Fresh transplant cycle	40.04 ± 7.74	–		
Freeze-thaw transplant cycle	108.18 ± 33.56	124.38 ± 41.51	1.023	0.311
Total TTP	52.34 ± 35.15	124.38 ± 41.51	-4.436	0.001
Embryo-free transfer cycle rate (%)	2.1 (4/191)	19.2 (65/339)	31.472	<0.001
Cost of ovulation promotion treatment ($)	3936.96 (2123.59, 4429.08)	3383.33 (702, 4921.20)	-1.058	0.290

*Fisher precision inspection.

### 3.4 Analysis of perinatal outcomes

There were no statistical differences between groups in perinatal outcomes for neonatal outcomes and pregnancy complications in Groups A1, A2, B1, and B2 (*p > 0.05*) (**Table 7**).

**Table 7:** Analysis of perinatal outcomes.

Projects	A1	A2	B1	B2	P *value*
Number of live births	12	57	95	121	
Premature babies	25.00 (3/12)	26.32 (15/57)	15.79 (15/95)	12.40 (15/121)	0.111
Low birth weight babies	33.33 (4/12)	12.28 (7/57)	11.58 (11/95)	14.88 (18/121)	0.238
Very low birth weight babies*	0.00 (0/12)	7.02 (4/57)	6.32 (6/95)	2.48 (3/121)	0.359
Hypertension in pregnancy*	0.00 (0/54)	1.87 (4/214)	3.14 (6/191)	3.24 (11/339)	0.570
Gestational diabetes	1.85 (1/54)	3.27 (7/214)	5.24 (10/191)	4.42 (15/339)	0.707
Placenta praevia	1.85 (1/54)	0.80 (1/214)	1.05 (2/191)	0.30 (1/339)	0.280
Premature rupture of membranes	0.00 (0/54)	5.65 (7/214)	2.09 (4/191)	2.95 (10/339)	0.648

* Fisher precision inspection.

## 4 Discussion

By competing with endogenous GnRH for pituitary cell GnRH receptors, GnRH antagonists regulate pituitary surface GnRH receptor levels, inhibit early LH peaks and endogenous FSH and LH levels, more closely resemble the development of follicles under normal physiological conditions, prevent early follicular luteinization or ovulation, and result in more high-quality eggs (12). PPOS (13) The European Society of Human Reproduction and Embryology (ESHRE) guidelines for ovarian stimulation in IVF/ICSI state that antagonist regimens are routinely preferred ovulation regimens in the POR population (14). A recent study by Du et al. in our center showed that (15) for patients with a low prognosis diagnosed according to the POSEIDON criteria, the CLBR of the PPOS regimen was comparable to that of the GnRH antagonist regimen. In contrast with the PPOS regimen, which supports fresh embryo transfer, the PPOS regimen must be combined with total freezing and delayed embryo transfer; therefore, the antagonist regimen significantly reduces the time to clinical pregnancy, consistent with the data from this trial, and the reduction in TTP may reduce patient anxiety to some extent (16).

Many studies have compared pregnancy outcomes between the antagonist and PPOS regimens. Chen et al. (16) randomized 340 patients eligible for Bologna with POR to a PPOS regimen versus an antagonist regimen to promote ovulation and found similar live birth rates for the PPOS and GnRH antagonist regimens. A recent meta-analysis showed that (17) the total number of Gn days, total number of eggs gained, clinical pregnancy rates after transplantation, persistent pregnancy rates, and miscarriage rates were similar in the PPOS and GnRH antagonist regimens. However, there are few studies on cumulative live birth rate. CLBR is a more comprehensive, rational, and objective method than traditional IVF pregnancy outcome statistics, based on pregnancies obtained after one controlled ovarian stimulation cycle of fresh embryos transferred and all subsequent frozen embryos allowed for transfer (18). The main observation of this study was the CLBR, and the subjects were women of reproductive age (< 35 years). The effects of antagonist and PPOS regimens on the CLBR and perinatal outcomes of young patients with different levels of low AMH were investigated. The results showed that higher AMH levels were associated with higher CLBR levels in younger patients with low AMH (AMH < 1. 2ng/mL) using the same ovulation induction regimen, no significant difference in the CLBR between the GnRH antagonist and PPOS regimens in young patients with very low AMH levels (AMH < 0. 5 ng/mL), and the GnRH antagonist regimen in young patients with low AMH (0. 5 ng/mL ≤ AMH < 1. 2 ng/mL)can increase the CLBR.

Since 2015, Kuang (19) et al. have proposed that progestins could be used as a low-cost alternative to GnRH analogs in the treatment of patients with POR, with MPA and dydrogesterone as the main progestins. Subsequently, Evans (20) et al. constructed a cost model for PPOS and GnRH antagonist regimens. If only fresh embryo transfer is considered, the antagonist progesterone cost is higher than that of PPOS, and is not economically advantageous. As the PPOS regimen has a frozen embryo policy only, assuming a certain frozen embryo cycle time and cost in the model setting, the PPOS regimen still has a higher economic benefit per live birth than the antagonist regimen. This result is consistent with the superior cost-effectiveness of the PPOS regimen in facilitating ovulation observed in this study. Due to the limited data on the experimental sample size, there were also confounding factors in the data screening and statistical calculations.

With the increasing maturity of IVF/ICSI technology, the rate of conceiving and deliveries using this technique is increasing, with more than 1 million babies born worldwide through assisted reproduction (21). However, the health of these children is a growing concern. To investigate the safety of the two protocols for offspring health, further analysis of perinatal outcomes and neonatal outcomes based on follow-up records was conducted, which showed no significant differences in perinatal outcomes among groups A1, A2, B1, and B2 (p > 0.05).

## Conclusion

In summary, for young patients with AMH < 0.5 ng/mL levels, the CLBR outcome of the GnRH antagonist program was similar to that of the PPOS program, which could shorten the time to clinical pregnancy, and the PPOS program was more economical and beneficial than that of the GnRH antagonist program. For young patients with low AMH levels of 0.5 ng/mL ≤ AMH < 1.2 ng/mL, the GnRH antagonist regimen may be more suitable for improving CLBR than the PPOS regimen and the perinatal outcomes of the two regimens are similar.

## Data availability statement

The original contributions presented in the study are included in the article/supplementary files. Further inquiries can be directed to the corresponding author.

## Ethics statement

Studies involving human participants were reviewed and approved by the Ethics Committee of the Third Affiliated Hospital of Zhengzhou University. Written informed consent for participation was not required for this study, in accordance with national legislation and institutional requirements.

## Author contributions

YG and ZL performed data extraction and analysis. KW, JZ, and BR reviewed the data. RJ and ZL drafted the manuscript. All authors contributed to the manuscript and approved the submitted version.

## Acknowledgments

We acknowledge the patients who participated in the study. We also thank the American Journal Experts for their professional manuscript editing services.

## Conflict of interest

The authors declare that the research was conducted in the absence of any commercial or financial relationships that could be construed as potential conflicts of interest.

## Publisher’s note

All claims expressed in this article are solely those of the authors and do not necessarily represent those of their affiliated organizations, or those of the publisher, the editors and the reviewers. Any product that may be evaluated in this article, or claim that may be made by its manufacturer, is not guaranteed or endorsed by the publisher.
